# Manipulation of Signaling Thresholds in “Engineered Stem Cell Niches” Identifies Design Criteria for Pluripotent Stem Cell Screens

**DOI:** 10.1371/journal.pone.0006438

**Published:** 2009-07-30

**Authors:** Raheem Peerani, Kento Onishi, Alborz Mahdavi, Eugenia Kumacheva, Peter W. Zandstra

**Affiliations:** 1 Institute of Biomaterials and Biomedical Engineering, University of Toronto, Toronto, Ontario, Canada; 2 Department of Chemical Engineering and Applied Chemistry, University of Toronto, Toronto, Ontario, Canada; 3 Department of Chemistry, University of Toronto, Toronto, Ontario, Canada; Virginia Tech, United States of America

## Abstract

*In vivo*, stem cell fate is regulated by local microenvironmental parameters. Governing parameters in this stem cell niche include soluble factors, extra-cellular matrix, and cell-cell interactions. The complexity of this *in vivo* niche limits analyses into how individual niche parameters regulate stem cell fate. Herein we use mouse embryonic stem cells (mESC) and micro-contact printing (µCP) to investigate how niche size controls endogenous signaling thresholds. µCP is used to restrict colony diameter, separation, and degree of clustering. We show, for the first time, spatial control over the activation of the Janus kinase/signal transducer and activator of transcription pathway (Jak-Stat). The functional consequences of this niche-size-dependent signaling control are confirmed by demonstrating that direct and indirect transcriptional targets of Stat3, including members of the Jak-Stat pathway and pluripotency-associated genes, are regulated by colony size. Modeling results and empirical observations demonstrate that colonies less than 100 µm in diameter are too small to maximize endogenous Stat3 activation and that colonies separated by more than 400 µm can be considered independent from each other. These results define parameter boundaries for the use of ESCs in screening studies, demonstrate the importance of context in stem cell responsiveness to exogenous cues, and suggest that niche size is an important parameter in stem cell fate control.

## Introduction

*In vivo*, embryogenesis is a highly orchestrated process involving the interaction of several signaling networks that produce local morphogenetic cues that determine cell fate and tissue organization[1]. Embryonic stem cells (ESCs) which are derived from the inner cell mass (ICM) of the embryo, are capable of recapitulating some of the early events of embryogenesis and have been shown to be capable of differentiation into many adult cell types *in vitro* [2]–[4]. However, this promising capability of ESCs is often offset by the fact that ESC cultures can be highly heterogeneous, over resulting in low yields of target cell types upon differentiation. This *in vitro* situation can be contrasted to embryogenesis where cell fate and spatial location appear tightly regulated. Motivated to address this disparity, we set out to quantitatively characterize the parameters that govern the spatially mediated activation of signaling and cell fate in mouse ESC (mESC) cultures using mathematical modeling and experimentation.

Recently, it has been shown that micro-patterning stem cells cultures in two- and three-dimensions can regulate typically uncontrolled ESC culture parameters such as colony size, distance between colonies, ECM substrate, and cell-cell interactions [5]–[7]. High-throughput platforms have also screened the effect of various extra-cellular matrix (ECM) and soluble growth factors on stem cell differentiation[8]–[11]. Despite the increase in use of micro-scale approaches to stem cell bioengineering, parameters which govern the design of micropatterned stem cell cultures, namely colony size and separation, have not been investigated for their effects on endogenous signaling, a parameter that could be important for the control of cell specification and for interpreting the effects of test conditions on pluripotent cell fate.

In this study, we investigate whether micro-patterning mESC cultures directly modulates paracrine signaling through the Janus kinase – signal transducer and activator of transcription (Jak-Stat) pathway. This pathway is activated by the *interleukin*-6 (IL-6) family of cytokines including leukemia inhibitory factor (LIF) and is typically required for the derivation and maintenance of mESCs *in vitro* [12]–[14]. Receptor-ligand binding results in the phosphorylation of the tyrosine-705 residue of signal transducer and activator of transcription 3 (pStat3) by receptor-associated Janus Kinases (Jaks), followed by pStat3 translocation to the nucleus[15]–[17]. Direct transcriptional targets of pStat3 include members of the Jak-Stat pathway, namely, gp130, Stat3, suppressor of cytokine signaling 3 (Socs3), and LIF receptor (LIFR)[18]. Pathway-associated targets include c-myc[19], Jumonji domain containing protein 1a (Jmjd1a)[20], [21], heterochromatin protein 1 (HP1)[22] and DNA methyltransferase 1 (DNMT1)[23], and indirect targets include pluripotency-associated core transcriptional network genes such as Oct-4[24], Nanog[25], Sox2[26], Kruppel-like factor 4 (Klf4)[27], and Sall-4[28]. Measurement of these targets provide a sensitive indication of the level of functional activation of the Jak-Stat pathway.

In previous studies, we have used *in silico* modeling and experimental observation to demonstrate that the gp130-Jak-Stat pathway acts in a positive feedback loop that controls transcriptional expression of LIF signaling components[18], [29]. This loop confers mESCs with a sensitivity towards the concentration of exogenous LIF such that differentiation-inhibitory and differentiation-permissive conditions occur over small changes in LIF concentration[18]. We now specifically test whether colony size-mediated control can be used to regulate Jak/Stat activation. Our approach was to first develop a mathematical model to predict how paracrine signaling thresholds are produced in uncontrolled ESC cultures and how they can be modulated by micro-patterning cultures. We then validated this model by measuring local pStat3 activation levels in both culture systems. Using the model, we were successfully able to predict how Stat3 activation is modulated by three micro-fabrication parameters: colony size, colony separation, and degree of clustering. Modeling results and empirical observations demonstrate that colonies less than 100 µm in diameter are too small to maximize endogenous Stat3 activation and that colonies separated by more than 400 µm can be considered independent from each other. These results define parameter boundaries for the use of ESCs in screening studies, demonstrate the importance of context in stem cell responsiveness to exogenous cues, and suggest that niche size is an important parameter in stem cell fate control.

### Model Development

Autocrine and paracrine signaling provide two means for cells to probe their micro-environment and communicate with other cells. In particular, autocrine loops have been proposed to act as a means of “cell sonar” whereby by cells can probe their microenvironment and respond based on the capture of self-secreted ligands[30]. Likewise, paracrine signals seem to be one of the primary means of induction during development[1]. The model described in this paper extends a stochastic model developed previously that predicts the fraction of autocrine and paracrine trajectories captured by a single cell in cell culture assays[31], [32]. In this previous work, a general solution to ligand lifetime and spatial trapping distribution is calculated based on the diffusivity of the ligand (*D*), single-cell parameters such as total receptor number (*R_t_*), cell radius (r_cell_), binding affinity (*k_on_* and *k_off_*), and the single-cell trapping efficiency (κ). Under the infinite media height model, the fraction of autocrine trajectories (*P_au_*) was shown to be independent of total cell density and medium height, while the trapping density of paracrine trajectories [*p(r)*] had radial (*r*) and total cell density (σ) dependencies (Fig. S1). The parameter *r* is the distance between cells. The major equations provided by the earlier modeling work are the capture probabilities of autocrine and paracrine ligands:
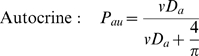
(1)

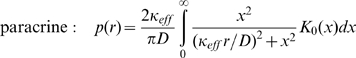
(2)where *D_a_* = κr_cell_/*D* is the Damkohler number (dimensionless reaction/diffusion ratio), K_o_(x) is the modified zeroth order Bessel function of the second kind which allows for *p*(r) to decay exponentially with increasing r, and *v* is the internalization ratio and is equal to k_e_/(k_e_+k_off_) where k_e_ and k_off_ and the first order rate constants for endocytosis and ligand-receptor dissociation respectively. 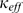 is the effective single-cell trapping efficiency for paracrine trajectories and is dependent upon cell density and the Damkohler number:
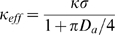
(3)


The rate constant, 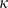, refers to the single cell trapping efficiency and is equal to:
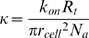
(4)


In this study, equations (1)–(4) were modified to incorporate the following features. First, receptor ligand complex number per cell (C_n_) is calculated for each cell by summing the autocrine trajectories with the paracrine trajectories from each of the other cells on the surface. Second, receptor number per cell (R_t_) is no longer constant but follows a Gaussian distribution amongst the entire cell population. Each cell is given a value for R_t_ between 300–700 receptors upon model initialization[33]. Third, complex degradation rate (*k_deg_*) is included as a parameter as it known that Jak-Stat pathway stimulation can induce lysosome-dependent receptor degradation[34], [35]. With these modifications in place, complex number for cell *i*,(*C_n_*)*_i,_* is calculated by summing the autocrine trajectories (P*^i^_au_*) with the paracrine trajectories from other cells on the surface (P*^i^_para_*). This sum is then multiplied by the ligand secretion rate (*v*_l_) minus the degradation rate (*k_deg_*) and simulation time (*t*) to obtain *(C_n_)i*:
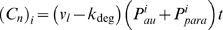
(5)

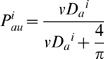
(6)

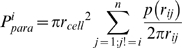
(7)

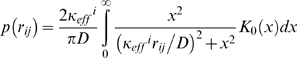
(8)


Note that D_a_ and κ_eff_ must be solved for each cell i because receptor number per cell is no longer constant. Thus, equations (1) and (2) have to be calculated with the corresponding receptor number to become equations (6) and (8) respectively.

The use of equations (5)–(7) requires additional assumptions not present in the original model that need to be highlighted. First, the spatial probability density of traps is assumed to be the same for a random configuration and an array of colonies at the same fractional coverage. This assumption is equivalent to saying that κ_eff_ remains constant for a cell population distributed randomly across the surface or “micro-patterned” into a single corner. Second, the complex degradation rate (*k_deg_*) is assumed to be constant with respect to time and independent of the number of complexes. Last, it is assumed that distances between cells can be segregated into 1 µm increments (i.e. 1/10^th^ cell width). This assumption is made to simplify the calculation of equation (8). Equation (8) is calculated in 1 µm increments for all distances ranging from the minimum and maximum cell separation distances, i.e. 2*r_cell_ and 2^1/2^*(culture width) respectively. This allows trapping density probabilities to be tabulated, saving computation time. The parameters for the simulation were chosen based upon previous work done on the gp130-Jak-Stat signaling pathway or in similar systems (Table 1).

**Table 1:** Parameters used in simulations.

Parameter	Description	Value	Unit	Ref.
R_t_	Total receptors per cell	300–700	#	[33]
r_cell_	Radius of a cell	10	um	Empirically determined
D	Diffusivity of LIF (actually IL-6)	2.7×10^−7^	cm^2^/s	[47]
k_on_	Association rate constant	0.2×10^9^	M^−1^min^−1^	[29]
k_off_	Dissociation rate constant	0.0011	min^−1^	[29]
k_e_	Endocytosis rate constant	0.0099	min^−1^	[29]
k_deg_	Degradation rate of complexes	0.2	min^−1^	[29]
v_l_	Ligand secretion rate	0.831	#/min	[48]
time	Simulation time	24	hrs	

## Results

### Theoretical prediction of endogenous signaling activation in non-patterned versus micro-patterning cell cultures

**Figure 1:**
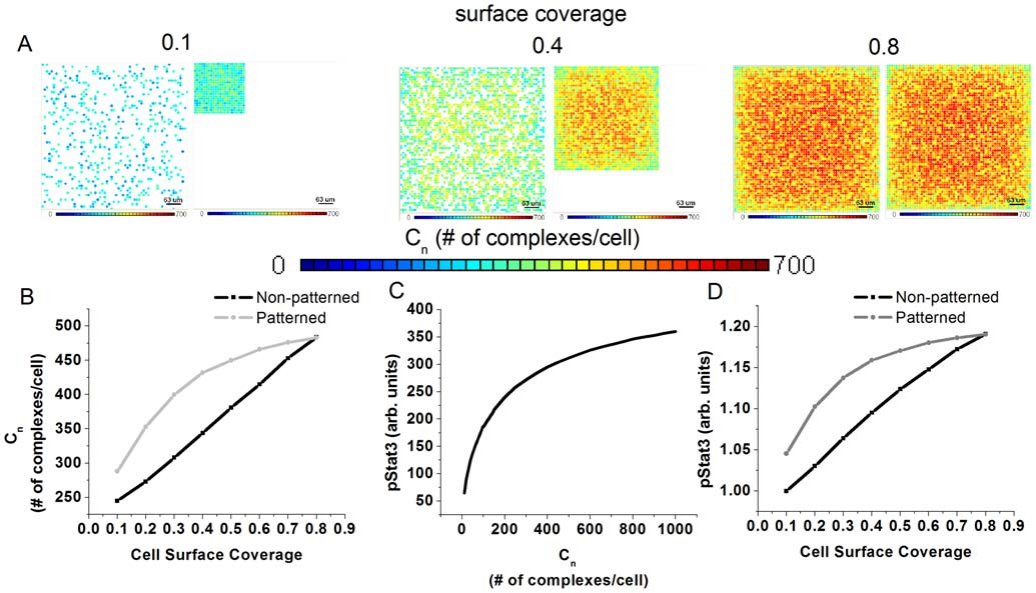
Theoretical prediction of how endogenous signaling activation increases upon micro-patterning cell cultures. A) Visual heat maps indicating the intensity and distribution of the number of bound ligand-receptor complexes (C_n_) as a function of cell surface coverage. Cell surface coverage is defined as the ratio between the area occupied by cells and the total cell culture area. As cell density or surface coverage increases, the average C_n_ increases. B) Quantification of the average C_n_ per cell as a function of cell surface coverage comparing the patterned and non-patterned cases. There is a window of opportunity between 0.1 and 0.8 cell surface coverage in which patterning will increase the average C_n_ per cell in a colony. C) Co-relation between C_n_ and the levels of nuclear pStat3 in a single cell as predicted by a previously published model[29]. D) Quantification of the predicted nuclear pStat3 accumulation in cells as a function of cell surface coverage.

As an initial step to determine the effect of micro-patterning on cell cultures, the average number of complexes per cell (C_n_) was calculated as a function of cell surface coverage under non-patterned and patterned conditions (Fig. 1). Cells were assumed to be flat disks with a radius of 5 µm and the cell culture area to be 0.3 cm^2^ (approximately the bottom of a well in a 96-well plate). Cell surface coverage was the ratio of the area occupied by cells to the total cell culture area. In the non-patterned case, cells were randomly given non-overlapping spatial co-ordinates until the appropriate cell surface coverage was attained. In the patterned case, cells were grouped into a square-packed arrangement keeping the cell surface coverage constant. C_n_ was then calculated for each cell in both spatial arrangements and visually represented using a heat map (Fig. 1A). The average C_n_ for each spatial arrangement was then calculated for cell surface coverages ranging from 0.1 (10% confluent) to 0.8 (80% confluent) (Fig. 1B). Interestingly, the random spatial configuration exhibited a linear increase in C_n_ with cell surface coverage while the micro-patterned arrangement had a logarithmic trend. This indicated that there is a window of opportunity for micro-patterning to affect C_n_ between 0.1 and 0.8 surface fractional coverage. The upper limit is intuitive as cultures upon reaching confluence effectively have the same local cell density as a patterned culture. Using a previously published model[29], a correlation between C_n_ and nuclear pStat3 accumulation was predicted (Fig. 1C). This plot was generated in order to validate the model using a metric that is easily measured experimentally, i.e. nuclear accumulation of pStat3 using quantitative immuncytochemistry. Lastly, the predicted nuclear accumulation of pStat3 as a function of cell surface coverage was obtained showing the same trends as C_n_ using the co-relation provided (Fig. 1D).

### Heterogeneity in endogenous Jak-Stat activation in mESC cultures can be predicted *in silico*

**Figure 2:**
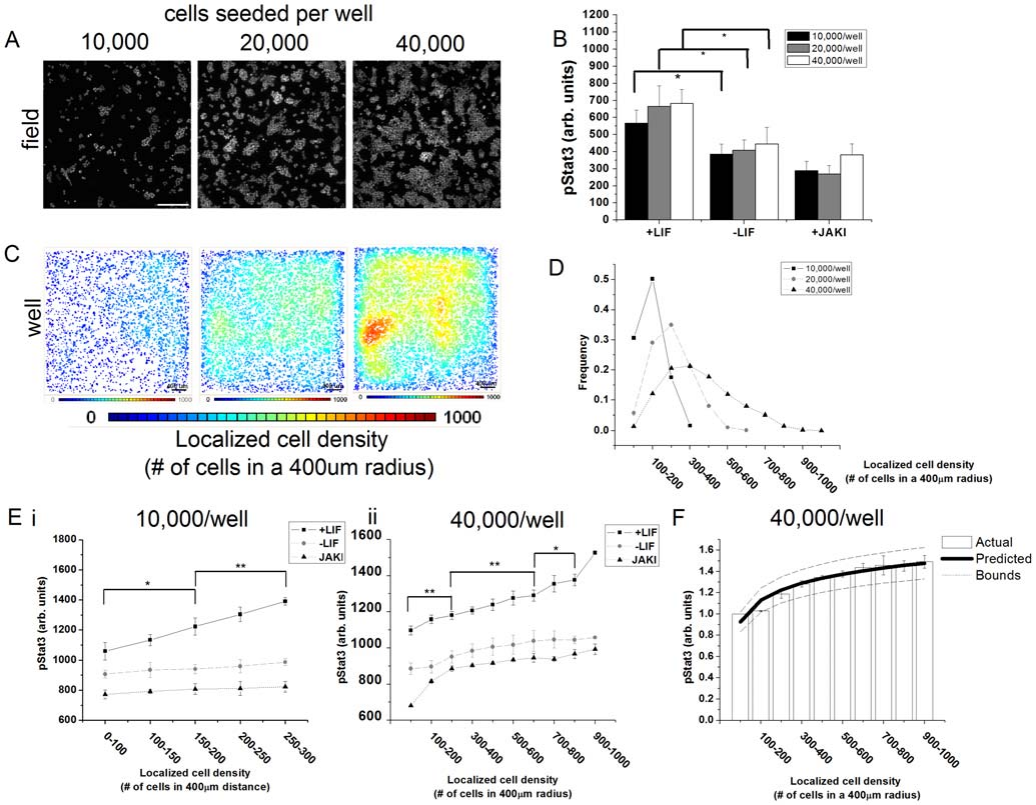
Model validation using the activation of the Jak-Stat signaling pathway in non-patterned mouse ESCs cultures. A) Single-field Hoechst 33342 micrographs of mESCs seeded at various densities in 96-well plates after being cultured for 24 hours in serum-free media without LIF. Scale-bar is 200 µm. B) Quantification of the average single-cell nuclear pStat3 accumulation after 24 hours of culture. While differences between exogenous supplementations were statistically significant at a given seeding density (p<0.05), no significance was present between cell seeding densities (p>0.40) for a given treatment. C) Heat maps indicating the increase and distribution in local cell density which is defined as the number of neighbouring cells within a 400 µm radius. The local cell density was calculated using an already published algorithm (See Materials and Methods)[46]. D) Histogram for the localized cell density as a function of initial seeding density showing that as initial cell density increases, the mean localized cell density increases and the distribution of cell densities broaden. E) Quantification of pStat3 signaling gradients present within single wells using the Neighbours Analysis algorithm. At low seeding densities, 10,000 cell/well (E–i), pStat3 can be seen to be increasing with cell density only in the presence of LIF whereas at a higher cell density, 40,000 cells/well (E–ii), signaling gradients can be found under all three treatments. Error bars represent the SEM of 9 wells cultures in three separate trials. F) Predicted and actual measurements of single-cell pStat3 levels as function of localized cell density demonstrating that the mathematical model developed in this study can be used to predict spatial fluctuations in signal activation in the Jak-Stat in mESCs. Data for this plot is taken for a single 96-well plate seeded at 40,000/well in the no LIF condition. While the percent increase in pStat3 in this figure is approximately 40%, the range in percent change is 10–40% for n = 9 wells. Error bars represent the SEM of at least 500 cells within each bin along the x-axis.

To demonstrate the ability of the mathematical model to predict endogenous activation of Stat3, mESCs were seeded at densities ranging from 10,000–40,000 cells per well in a 96-well plate and cultured for 24 hrs. Cells were cultured in serum-free media containing LIF, no LIF, and 600 nM Jak inhibitor (JakI) to inhibit the pathway. The cells spontaneously produced colonies with an average size that depended upon the initial seeding density (Fig. 2A). The average single-cell pStat3 activation was calculated for each treatment and seeding density (Fig. 2B). Statistical significance (p<0.05) was found between treatment conditions for a given cell seeding density. However, no significance was found (p>0.4) between cell seeding densities for a given treatment. As a quantitative metric of the microenvironment, the local cell density, defined as the number of neighbours cells within a 400 µm was calculated using a previously developed algorithm, Neighbours Analysis (Supplementary Fig. S2). By using this algorithm, heat maps (Fig. 2C) and histograms (Fig. 2D) were generated to analyze the variance in local cell density in each well. Mean localized cell density increased with higher seeding densities as expected. However, the histograms also indicate that increasing seeding density leads to a broader distribution of localized cell densities across the well. Such an observation suggests that one can attempt to increase paracrine signaling by simply seeding more cells into the well, however, such an action will simultaneously increase the heterogeneity within the well by creating multiple local microenvironments consisting of a differing number of cells.

Consequently, after measuring nuclear levels of activated Stat3 (pStat3) at the single cell level, no statistically significant increase in pStat3 was found as a function of initial cell density (cells/well). Conversely, if the localized cell density is incorporated into the measurement such that the single cell nuclear pStat3 levels are averaged across all cells having the same localized cell density, statistically significant increases in pStat3 as a function of localized cell density could be found (Fig. 2E, i and ii). Furthermore, the increase in pStat3 can be predicted by inputting the spatial arrangement of cells into the mathematical model (Fig. 2F). Note that without including information about the local cell density of a cell, simple quantitative immunocytochemistry would yield the erroneous conclusion that cell density does not affect pStat3 activation. The above analysis suggests that it is indeed the local microenvironment of a cell, rather than the well's macroscopic environment, that correlates with endogenous signal activation of a single-cell. Moreover, the effect of the local microenvironment can be adequately described by the mathematical model provided.

Interestingly, gradients in pStat3 were greater in the presence of supplemental LIF (500 pM) compared to non-LIF supplemented cells (Figure 2E), suggesting that pStat3 activation is not saturated upon LIF addition. In previous work, it has been demonstrated that there is a non-LIF autocrine factor that signals through the gp130 receptor family that regulates pStat3 activation in the presence of 500 pM LIF[36]. Furthermore, it has been shown that signaling in the gp130-Jak-Stat pathway in mESCs exhibits a switch-like response to LIF, as a consequence of a positive feedback loop that controls transcription of signaling pathway components[18], [29]. The effect of this autoregulatory behaviour is that sufficiently high exogenous LIF maintains pathway responsiveness, i.e. the “on” state, whereas low concentration or no exogenous LIF cause ESCs to adopt a state of weak responsiveness, i.e. “off” state. Thus, the smaller absolute gradients in pStat3 seen in conditions without LIF may be due to the down-regulation of pathway components due to decreased responsiveness to the pathway.

To interrogate this possibility, we examined the sensitivity of pStat3 gradients to the changes in expression of gp130-Jak-Stat pathway components *in silico*. Three model parameters were varied: endogenous ligand secretion, receptor number (Rt), and the introduction of an autoregulatory positive feedback loop that increased Stat3 (Supplementary Fig. S3) {Davey, 2007 #23}. Note that the feedback loop was incorporated into this model by re-calculating pStat3 as a function of C_n_ as originally shown in Figure 1D but with positive feedback. These *in silico* experiments suggests that the relative changes in pStat3 gradients with respect to localized cell density will increase in the presence of the postitive feedback loop. As exogenous LIF has been shown to increase endogenous gp130 ligand production and receptor number, only the autoregulatory positive feedback loop can account for the relative increase in pStat3 with local cell density [18]. An increase in the other two variables, ligand secretion and receptor number, would decrease these relative changes (not observed). Consequently, the differences in the pStat3 gradients observed between the 500 pM LIF and no LIF conditions can be attributed to the amount of Stat3 in the cell as regulated by the autoregulatory feedback loop present in mESCs rather than extra-ceullar ligand availability or receptor number.

### Restricting colony diameter controls the activation of the Jak-Stat pathway

**Figure 3:**
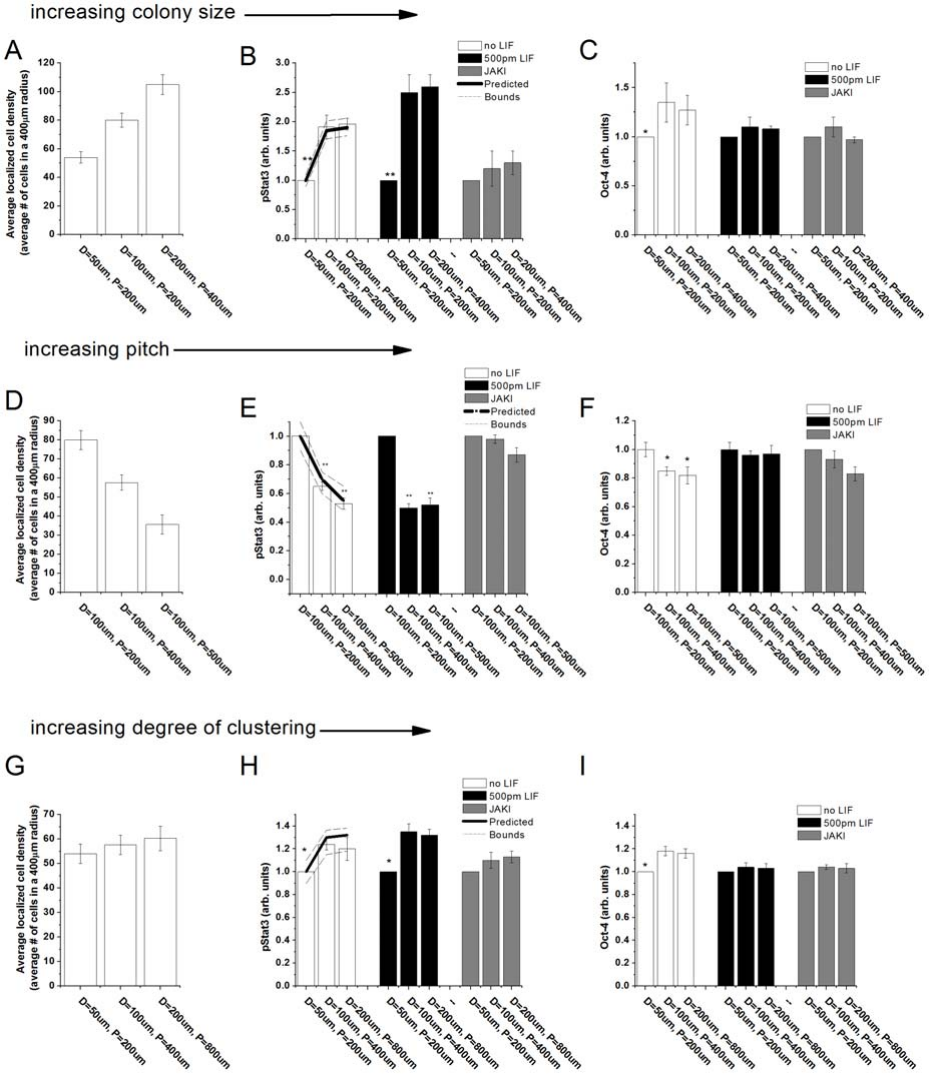
Modeling predicts the endogenous activation of Stat3 as regulated by the spatial organization of mESC cultures. In the first experiment (A–C), mESCs were cultured in colonies with diameters (D) ranging from 50–200 µm. Pitch (P), the centre-to-centre distance between colonies, was kept constant for the two smaller colonies and was increased to accommodate the larger D = 200 µm colony. A) Average local cell density calculations for each pattern type demonstrating the co-relation between colony diameter and the local cell density. B) Predicted and actual measurements of single-cell pStat3 levels as function of increasing colony diameter. White bars indicate cultures with no LIF supplementation, black bars with 500 pM LIF supplementation, and grey bars with 600 nM JAK inhibitor. C) Experimental measurements of Oct-4 intensity demonstrated the co-relation between colony diameter, pStat3, and Oct-4 expression with LIF deprivation. In the second experiment (D–F), mESCs were cultured in colonies with fixed diameter, D = 100 µm, while pitch (P) ranged from 200-500 µm. D) Average local cell density calculations for each pattern type demonstrating the inverse co-relation between pitch and the local cell density. E) Predicted and actual measurements of single-cell pStat3 levels as function of decreasing pitch. F) Experimental measurements of Oct-4 intensity demonstrated the inverse co-relation between pitch and Oct-4 expression. In the last experiment (G–I), both colony diameter and pitch were altered to increase the degree of clustering while keeping the total available area for seeding constant. G) Average local cell density calculations for each pattern type demonstrating that the local cell density was within experimental error between pattern types. H) Predicted and actual measurements of single-cell pStat3 levels as function of degree of clustering. I) Experimental measurements of Oct-4 intensity demonstrating a positive co-relation between pStat3 and Oct-4. Representative bright field images of micro-patterned mESC cultures can be found in Supplementary Fig. S4.

The preceding analysis suggests that endogenous activation of Stat3 for a single cell correlates with the localized cell density of its immediate microenvironment. This observation allows for hypothesis that micro-patterning mESCS which provides control over the local cell density by restricting colony diameter (D), separation (pitch, P), and degree of clustering, can modulate endogenous Stat3 activity. To test this hypothesis, mESCs were patterned in colony sizes ranging from 50–200 um with pitch ranging from 200–400 um (Supplementary Fig. S4), in a serum-free media with and without 500 pM exogenous LIF supplementation and the average local cell density was obtained for each patterned culture (Fig. 3A). The coefficient of variance in the distribution of local cell densities in micro-patterned colonies was found to be lower than in the non-patterned cultures. Once again, pStat3 activation in these micro-patterned colonies was predicted accurately by the model (Fig. 3B). Oct-4 expression in ESCs correlated with pStat3 activation and colony diameter (Fig. 3C), indicating that even after only 24 h of culture cells are already responding to the signaling gradients established using our spatial segregation strategy. Differences in pStat3 activation were accentuated with 500 pM LIF supplementation (black bars) and diminished in 600 nM JAKI. This data suggests that the autoregulatory postitive feedback loop maintained by exogenous LIF is maintained in micropatterned cultures and are JAK dependent. Differences in Oct-4 levels amongst micro-patterned colonies were minimal with exogenous LIF supplementation and JAKI.

### Increasing pitch decreases activation of the Jak-Stat pathway

We next wanted to demonstrate the effect of increasing colony separation (pitch) on endogenous Stat3 activation. MESCs were cultured with colony diameters fixed to D = 100 um and pitch ranging from P = 100–500 um. As expected, Neighbours Analysis indicated that as pitch increased the local cell density decreased (Fig. 3D). Nuclear pStat3 values were inversely proportional to pitch and were successfully predicted by the model (Fig. 3E). Likewise, Oct-4 expression decreased with increasing pitch. Exogenous LIF supplementation and JAKI had the same effect on pStat3 and Oct-4 as the previous micro-patterning experiment. Interestingly, there is no statistically significant difference in pStat3 from P = 400 um to P = 500 um suggesting that the colonies are independent of each other at this distance with respect to diffusible ligands that activate Stat3 as a downstream effector (Fig. 3F). Notably, the only extrinsic parameter of the culture changing in these experiments is pitch. Critically, the number of cell-cell contacts and distribution of mechanical forces within a colony can be presumed to be constant. As phenotypic changes in micro-patterned cultures could be attributed to either to a change in receptor-ligand binding, the number of cell-cell contacts, or distribution in mechanical forces, this experiment serves as an important control by keeping the latter two variables constant. Thus, this experiment demonstrates that micro-patterning can alter soluble ligand availability and binding.

### Increasing degree of clustering increases the activation of the Jak-Stat pathway

The last micro-fabrication parameter interrogated was degree of clustering. This experiment was conducted to further isolate the effect of spatial organization independent of any effects of overall cell density. In this study, colony diameter ranged from D = 50–200 µm and pitch from P = 200–800 µm, while keeping overall seeding area (and cell number) constant between pattern arrangements. The local cell density was calculated, using Neighbours Analysis, to be within experimental error for all three pattern types (Fig. 3G). With no LIF supplementation, levels of pStat3 increased with degree of clustering which was predicted by the model (Fig. 3H). Furthermore, Oct-4 expression increased with degree of clustering (Fig. 3I). As seen before, 500 pM LIF supplementation increased the relative change in pStat3 amongst micropatterns however, Oct-4 did not change significantly as Stat3 levels are above the differentiation threshold. JAKI had a similar effect as before by diminishing the observed differences. Note that increasing the degree of clustering was not as effective as doubling colony size in regulating pStat3 levels. These latter experiments, nonetheless, provide further support that the micro-organization of a culture can regulate the activation of an endogenous signaling pathway since the number of cells in the well and local cell density in each pattern type was approximately the same. Note that even though the local cell densities were similar, the average separation between cells would decrease as degree of clustering increases. This experiment demonstrates that paracrine activation of the Jak-Stat pathway is both cell-number and cell-separation dependent.

In order to reveal localized intra (within)-colony effects in the activation of the Jak-Stat pathway additional analysis was on performed on the D = 50 um and D = 100 um colonies (Supplementary Figure S5). This analysis demonstrated a radial dependence in pStat3 but not Oct-4. This data suggests that internal cells have increased activation of Stat3 relative to outer cells. Relative changes in Oct-4 may not be apparent, likely due to short-time frame of the experiment (24 hours); loss of pStat3 responsiveness has been shown to precede the loss of Oct-4[18].

### Micro-patterning mESC cultures regulates transcription of known direct and indirect Stat3 targets

**Figure 4:**
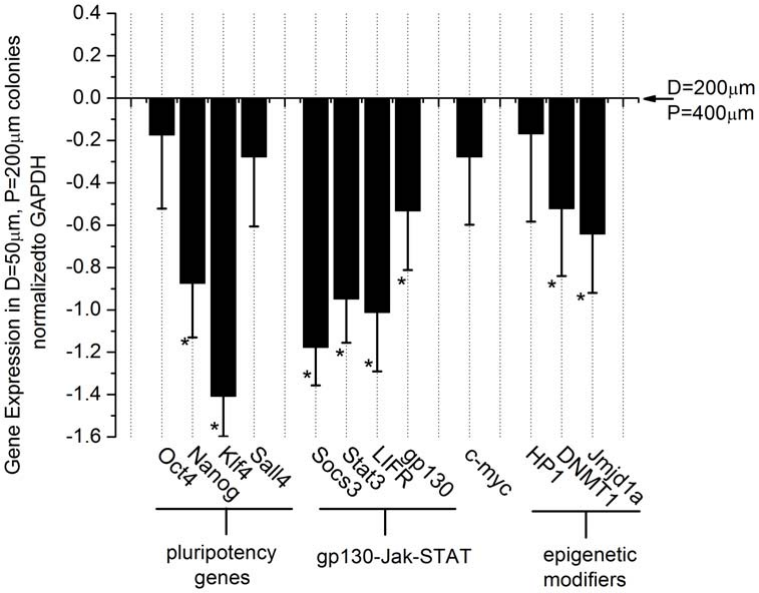
Micro-patterning mESC cultures regulates transcription of known Stat3 targets. Quantitative real-time PCR results of mESCs cultured on D = 50 µm, P = 200 µm and D = 200 µm, P = 400 µm pattern cultured for 24 hours in serum-free media without LIF. The data is normalized to the house-keeping gene GAPDH and each gene is plotted relative to its expression found in the D = 200 µm, P = 400 µm pattern. Pluripotency-associated genes, Oct-4, Nanog, Klf4, and Sal4 decrease in expression with smaller colony size. Likewise, members of the Jak-Stat pathway, including LIF receptor (LIFR), gp130, Stat3, and Socs3 all decrease with smaller colony size demonstrating spatial control of this auto-regulatory pathway. C-myc, another target of pStat3 is also down-regulated in small colonies. Epigenetic modifiers of mESCs including HP1, Dmnt1, and Jmd1ja are also down-regulated in small colonies. Error bars represent the S.E.M for n = 3 biological replicates. Asterisks indicate statistical significance of p<0.05 by the t-test.

After establishing that micro-patterned ESCs exhibit different signaling levels of Stat3 activation, we next sought to determine if this change in signaling activation had downstream consequences on known and predicted targets of Stat3. We hypothesized that Stat3 targets would be differentially expressed in small (D = 50 µm, P = 200 µm) colonies versus large (D = 200 µm, P = 400 µm) colonies because of the lower levels of pStat3 in small colonies. Quantitative real-time PCR (qRT-PCR) was used to quantify the expression of several possible targets of Stat3 (Fig. 4). As expected the pluripotency genes decreased with smaller colony size. The expression of Jak-Stat pathway members decreased as well in small colonies, an observation which is to be expected by the auto-regulatory behavior of this pathway as revealed previously in non-patterned cultures[18]. Genes related to the epigenetic status of mESCs including Jmid1a, Dnmt1, and HP1 decreased as well. These findings provide further evidence that spatial control over Stat3 and its downstream transcriptional targets can be achieved in micro-patterned mESC cultures with implications to the pluripotency network and epigenetic state of the mESCs.

## Discussion

**Figure 5:**
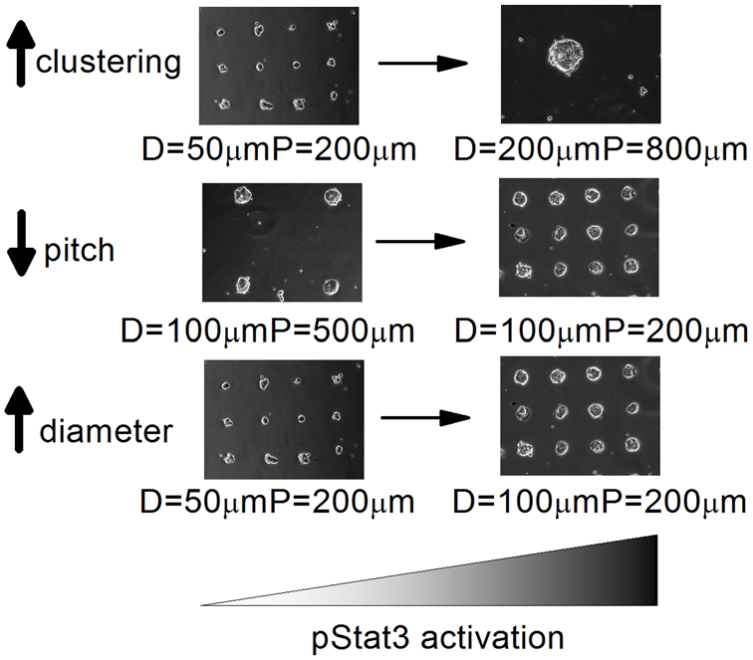
Micro-patterning mESC cultures provides spatial control over endogeneous Jak-Stat activation. Using *in silico* models and experimental validation, we have demonstrated that endogenous activation of the Jak-Stat pathway can be regulated spatially by micro-patterning mESC cultures. Three parameters were explored: increasing colony diameter, decreasing colony pitch, and increasing the degree of clustering.

Local gradients of activated signaling molecules exist in ESC cultures and these gradients correlate with the expression of pluripotent stem cell markers such as Oct-4 and Nanog. These gradients can be correlated to the localized cell density in a single well as well as manipulated directly using micro-patterning technologies. Specifically, by altering spatial arrangement, we have shown for the first time that altering colony diameter, the distance between colonies, and the degree of clustering of a culture can modulate in a predictive manner the nuclear levels of pStat3 in mESCs (Fig. 5).

To date, micro-scale approaches to modulating cell-ECM interactions and cell-cell contacts have provided insight into how *in vitro* niches can regulate stem cell fate. For instance, micro-patterning single adult mesenchymal stem cells on defined ECM substrates has been shown to provide inductive cues to regulate differentiation into osteoblasts or adipocytes through regulation of cytoskeleton tension[37]. Likewise, patterning mESCs in bow-tie micro-wells has demonstrated the effect of cell-cell interactions on neuroectoderm differentiation, perhaps through a mechanism involving connexin-43[38]. Interestingly, another study that cultured hepatocytes on micromachined silicon substrates capable of regulating cell-cell contacts dynamically demonstrated that both cell-cell contact and newly identified paracrine factor were required to retain the hepatocyte phenotype[39]. The activity of this paracrine factor, similar to the Stat3 activity demonstrated here, was limited to <400 µm. Thus, it is likely that the mathematical model and experimental approach used in this work are applicable to a wide range of signaling pathways in multiple cells types.

Modeling using finite element analysis has been used to predict the effects of micro-patterning on multi-cellular aggregates[40]. This previous work has revealed that regions of higher actin cytoskeleton tension can appear at edges of colonies leading to proliferative foci of endothelial cells. The mathematical model presented here complements this study by providing a model capable of describing soluble paracrine activity on micro-patterned multi-cellular aggregates. Developing *in silico* tools such as these to predict how cellular behaviour changes as a function of spatial organization will allow for mechanism-based design of lab-on-chip devices to regulate stem cell fate.

In this work, we have shown that small mESC colonies (D = 100 µm) are independent of each other at a distance of 400–500 µm and that D = 100 µm colonies are sufficiently large to maximize endogenous Stat3 activation. These observations can be explained by the manipulation of the diffusion and binding of locally secreted cytokines of the IL-6 family, as well as the responsiveness of mESCs to these ligands[36]. We have previously associated the secretion and responsiveness to IL-6 ligands to a fixed-location independent auto-regulatory niche (FLIAN) produced by mESCs to temporarily maintain self-renewal in the absence of LIF. MESCs are known to express several members of the IL-6 family of cytokines including IL-6, IL-11, LIF, oncostatin m, cardiotrophin-1 (CT-1), cardiotrophin-like cytokine (CLC), and ciliary neurotrophic factor (CNTF). These factors may contribute to the paracrine activation of the Jak-Stat pathway to maintain mESCs. Our results show that maximizing paracrine activation of endogenous signals cannot simply be achieved by seeding more cells in well without increasing the heterogeneity of a culture. Micro-patterned cultures regulate endogenous signaling without this side-effect.

Indeed, there are two critical differences between non-patterned and micropatterned cultures with respect to increased paracrine signaling. The first difference is that the model predicts a greater change in complex number (C_n_) and pStat3 under a wide range of cell densities when cells are patterned (logarithmic trend) versus non-patterned (linear trend) (Figure 1B and 1D). The reason for these trends is likely due to the increased cell clustering that occurs when cells are patterned, i.e. at the same local cell density cells in a micropatterned colony are all together whereas in non-patterned cultures non-uniformities occur. The second difference is that the non-uniformity in non-patterned seeded cultures leads to a systemic error in measurement. As the culture arrangement moves from a non-patterned to patterned spatial arrangement, several factors change including colony size, separation, and degree of clustering. In the case of Figure 2, the output (pStat3) represents the average over all these different microenvironments leading to a systemic error in the measurement. Consequently, the differences between cells would be under-represented. Indeed, the differences in paracrine signaling within non-patterned versus patterned cultures highlights the importance of our approach or other micro-patterning technologies in teasing apart these variables in the local microenvironment.

Interestingly, paracrine signaling of bone morphogenetic protein 2 (BMP2) has been implicated in regulating the niche-size of hematopoietic stem cells *in vivo* (HSCs)[41]. Moreover, other studies have shown that ectopically regulating the quantitative levels of Oct-4 can be used to induce trophectoderm and primitive mesoderm/endoderm[42]. Thus, the methodologies developed in this work allow for the systematic prediction, detection, and manipulation of endogenous signaling gradients *in vitro* along similar mechanisms found *in vivo* in order to regulate cell fate.

## Materials and Methods

### Micro-contact printing of ECM onto tissue culture substrates

Poly(dimethylsiloxane) (PDMS) stamps with feature sizes of 50–200 µm (D, diameter) and distance between features between 200–800 µm (P, pitch) were fabricated using standard soft lithography protocols provided elsewhere. The micro-contact printing protocol was based upon a technique detailed in other work[43]. Briefly, PDMS stamps were inked with a solution of 25 µg/mL of bovine fibronectin and 50 ug/mL of bovine gelatin prepared in sterile ddH_2_O for 1 hr. After inking, stamps were rinsed thoroughly in sterile ddH_2_O and dried with N_2_ gas. The stamps were then placed quickly onto tissue culture-treated plastic slides and placed in a humidity chamber with a relative humidity between 55–70% for 10 min. Silicone gaskets, 20 mm in diameter and 2.5 mm in height, were placed around the patterned regions to create a leak-proof well. Slides were passivated in 5% Pluronic ™ F-127 for 1 hr to prevent non-specific attachment.

### Cell Culture

R1 mouse embryonic stem cells were maintained at 370 C in humidified air with 5% CO2 in ESC culture medium comprised 80% Dulbecco's Modified Eagle Medium (DMEM, Gibco-BRL, Rockville, MD) supplemented with 15% ESC qualified fetal bovine serum (FBS), 100 U/mL (Gibco-BRL), 50 ug penicillin-streptomycin (Gibco-BRL), 2 mM L-glutamine (Gibco-BRL), 0.1 mM 2-mercaptoethanol (Sigma, St. Louis, MO), and 500 pM leukemia inhibitory factor (LIF, Chemicon, Temecula, CA). Culture flasks (Sarstedt, Newton, NC) were prepared prior to cell seeding by coating with a solution of 0.2% bovine gelatin (Sigma) in phosphate buffered saline (PBS, Gibco-BRL). All ESCs were used between passages 15–30. Under experimental conditions, the above media was used with 15%KNOCKOUT™ serum replacement (KOSR, Invitrogen) as a substitute for 15%FBS.

### Seeding of mESCs on non-patterned substrates

Single cell suspensions were generated by incubating with 0.25%trypsin with 1 mmEDTA (Trypsin-EDTA, Gibco-BRL) for 3 minutes and then quenching with ESC culture media. Cells were then resuspended in ESC culture media with 500 pM LIF containing 15%KOSR instead of 15%FBS. Cells were re-suspended at various concentrations ranging from 10000–40000 cell per well (volume/well = 200 uL). Cells were seeded into a tissue-culture treated 96-well plate coated overnight at 37°C with 25 ug/mL of bovine fibronectin and 50 ug/mL of bovine gelatin in ddH2O. Five replicates per concentration was used. Cells were seeded in KOSR-based media with LIF for 4 hrs, after which media was replaced with KOSR-based media with or without 500 pM LIF or 600 nM of Jak inhibitor 1 (Calbiochem). Cells were fixed and stained 20 hrs after media exchange.

### Culture of mESCs on non-patterned substrates

Single cell suspensions were generated by incubating with 0.25%trypsin with 1 mmEDTA (Trypsin-EDTA, Gibco-BRL) for 3 minutes and then quenching trypsin with ESC culture media. Cells were then resuspended in ESC culture media with 500 pM LIF containing 15%KOSR instead of 15%FBS. Cells were re-suspended at various concentrations ranging from 10000–40000 cell per well (volume/well = 200 µL). Cells were seeded into a tissue-culture treated 96-well plate coated overnight at 37°C with 25 µg/mL of bovine fibronectin and 50 µg/mL of bovine gelatin in ddH2O. Five replicates per concentration was used. Cells were seeded in KOSR-based media with LIF for 4 hrs, after which media was replaced with KOSR-based media with or without 500 pM LIF or 600 nM of Jak inhibitor 1 (Calbiochem). Cells were fixed and stained 20 hrs after media exchange.

### Culture of mESCs on patterned substrates

Single cells suspensions were created in a similar manner as the non-patterned case at a density of 2.50×10^5^ cells/well (volume = 750 uL) and incubated with the patterns for 4 hrs. The cells were washed with KOSR-based media without LIF three times to remove non-attached cells and then incubated in KOSR-based media without LIF for an additional 20 hrs. The seeding density chosen was empirically determined to ensure that the colonies were confluent and still a monolayer 24 hours later.

### Quantified immunohistochemistry of patterned and non-patterned substrates

Patterned and non-pattered cultures were fixed in 3.7% formaldehyde in PBS for 15 min at 37°C and were washed three times with PBS. Samples were then permeabilized in 100% methanol for 2 min, washed three times with PBS and incubated with blocking buffer consisting of 10%FBS in PBS overnight at 4°C. Samples were then stained with mouse anti-Oct-4 primary antibody (1∶200) and rabbit anti-pStat3 primary antibody (1∶200) diluted blocking buffer overnight at 4°C. Cells were washed 3x with PBS and incubated with goat anti-mouse IgG Alexafluor 488 secondary antibody (1∶200), goat anti-rabbit IgG Alexafluor 647 secondary antibody (1∶200), and Hoechst 33342 (0.1 ug/mL) to identify all nuclei. After staining, samples were imaged using the Cellomics Arrayscan V^TI^ high-throughput fluorescence microscope using the Target Activation algorithm to provide quantified image analysis of the spatial location of the centroid of the nucleus relative to the centre of the image as well as fluorescence intensity measurements for pStat3 and Oct-4 in the nucleus. To get colony area and the number of cells per colony, the Morphology Explorer™ image analysis algorithm available through the ArrayscanV^TI^ software was used.

### Calculation of the local cell density using Neighbours Analysis

Neighbours analysis is a set of algorithms developed in the Python™ scripting language used to calculate the number of neighbours a cell has within an arbitrary radius as well as the average distance of those neighbours to that cell (Fig. S2). The input to the algorithm is the spatial location of the nucleus relative to the centre of the image as provided by the Cellomics Target Activation Algorithm. Any software which gives the x-y coordinates and single-cell quantification of fluorescence could be used. Based on systems specifications of the microscope, well size, and the magnification of the objective lens, the program re-constructs the entire well. For every cell, the Euclidean distance between itself and all the other cells in well was calculated and the number of immediate neighbours was gated upon neighbours cells that were found within an arbitrary radial threshold of 400 µm. The number of neighbours within a 400 µm is the *localized cell density* of the cell. This threshold was found empirically by plotting nuclear pStat3 as a function of radial thresholds from 100–1000 µm and choosing the highest radial threshold that had a significant co-relation (R^2^). These radial thresholds provide an approximate size to the stem cell niche in spatial terms by suggesting that cells within a certain proximity are those that are most effective in communicating with a cell and thereby influencing its fate.

### Quantitative RT-PCR analysis

RNA from patterned mESC cultures was isolated using TRIzol reagent (Invitrogen) and purified including DNA digestion using the RNEasy Mini Kit (Qiagen) according to the manufacturers' protocols. cDNA synthesis was carried out using the Superscript First Strand Synthesis system (Invitrogen). Reaction conditions for the PCR reaction were incubation at 94°C for 10 min, followed by 40 cycles of 94°C for 30 s, 60°C for 30 s, and 72°C for 30 s using the Applied Biosystems 7900HT Fast Real-time PCR System. A table of the primers used can be found in the Table S1 [44], [45].

### Model Code

The entire model code was programmed in the Python scripting language (http://www.python.org). Visualization of gradients using chloropleth maps was implemented using the Python Imaging Library (PIL).

### Statistical Analysis

All data are reported as mean±standard deviation. Experiments measuring differences between means were assessed using paired Student's *t*-test with * indicating p<0.05 and ** indicating p'0.01. Experiments testing variances were assessed using the f-test.

## Supporting Information

Table S1 Primers used in this study.
(0.03 MB DOC)

Figure S1 Schematic of the spatial parameters of the model. Complex number for cell *i* (*C^i^n*) is proportional to the sum of the probabilities of ligand capture by autocrine trajectories (*P^i^au*)and paracrine trajectories (*P^i^para*) that are dependent of the radius of the cell (*r_cell_*). *P^i^para* is determined by summing the paracrine contributions of each cell pair in the well *p(r_ij_)*which has radial (*r_ij_*) and cell density (σ) dependencies.
(0.04 MB TIF)

Figure S2 Development of the Neighbours Analysis algorithm. A) Photo-micrographs at 10X of non-patterned mESCs immunostained with Hoechst 33342, Oct-4, and pStat3 and the resulting heat-map constructed using the Python Imaging Library (PIL). Masks around individual nuclei were drawn using the Target Activation algorithm. B) Schematic of the Neighbours Analysis algorithm which counts the number of cells within a 400 µm radius which is dubbed local cell density. C) Example of binning for cells seeded at a density of 40,000 cells per well. D) Representative pStat3 histograms for cells after binning. E) Summary plot for data taken from the histograms to illustrate co-relations between pStat3 and local cell density.
(1.54 MB TIF)

Figure S3 Theoretical predictions of how Jak-Stat pathway signaling components affect local signaling gradients. A) In these in silico experiments, the spatial arrangement of mESCs cultured in a single 96-well (approximately 35,000 cells) were inputted to the model. The predicted increase in pStat3 with increased localized cell density was computed while varying three parameters: endogenous ligand secretion, receptor number (*R_t_*), and the presence of a positive feedback loop that increases total Stat3 (C). A) Predicted gradients in Stat3 as a function of increasing ligand secretion. According to model data, the relative changes in Stat3 that co-relate with localized cell density decrease moderately with increased ligand secretion. B) Predicted gradients in Stat3 as a function of increased receptor number (*R_t_*). Increasing receptor number decreases the relative change in Stat3 with localized cell density. C) Predicted gradients in Stat3 in the presence of an auto-regulatory positive feedback loop that increases Stat3 in mESCs. This feedback loop has been previously described and modelled[18], [29]. The effect of this loop is to accentuate the effect of localized cell density on Stat3 gradients present in the culture.
(0.40 MB TIF)

Figure S4 Bright field images of micro-patterned colonies. Bright field images of mESCs seeded in six different pattern types after 24 hours of culture in serum-free media without LIF. Scale-bar is 200 µm.
(1.15 MB TIF)

Figure S5 Radial organization of pStat3 within colonies. To reveal intra-colony variation in protein expression or signal activation, single-cell pStat3 and Oct-4 expression was plotted as a function of distance from the centre of the colony. mESCs were patterned in D = 50 µm, P = 200 µm and D = 100 µm, P = 200 µm arrangements. A) A radial dependence in pStat3 was observed in in both D = 50 µm and D = 100 µm colonies. B) No radial depense in Oct-4 was observed suggesting that the timeframe of the experiment (16 hours) was too short to reveal significant changes in Oct-4 expression.
(0.10 MB TIF)
