# Supplementary material for: Manipulation of Signaling Thresholds in “Engineered Stem Cell Niches” Identifies Design Criteria for Pluripotent Stem Cell Screens
Source: PLoS One. 2009 Jul 30;4(7):e6438. doi: 10.1371/journal.pone.0006438 (PMC2713412; doi:10.1371/journal.pone.0006438)
Supplement: Table S1 — Primers used in this study. (0.03 MB DOC) [file pone.0006438.s001.doc]

**Supplementary Table S1. Primers used in this study.**

| ***GENE*** | **FORWARD PRIMER** | **REVERSE PRIMER** |
| --- | --- | --- |
| *C-myc* | TCT CCA TCC TAT GTT GCG GTC | TCC AAG TAA CTC GGT CAT aCAT CT |
| *Stat3* | TGG CAC CTT GGA TTG AGA GTC | GCA GGA ATC GGC TAT ATT GCT |
| *gp130* | ATC AGA GTG GGC AAC AGA GAA | TGA GGA GAC CTT CCC AAG GG |
| *Lif-R* | ACC TCC TCC TTA CTA CTG AAG TG | TTC CGT CCT TGG ATT CTG TAG A |
| *Klf4* | TCC TTT CCA ACT CGC TAA CCC | CGG ATC GGA TAG CTG AAG CTG |
| *Sall4* | CCC TGG GAA CTG CGA TGA AG | TCA GAG AGA CTA AAG AAC TCG GC |
| *Socs3* | CAA GAA CCT ACG CAT CCA GTG | CCA GCT TGA GTA CAC AGT CGA A |
| *Oct4* | AGT TGG CGT GGA GAC TTT GC | CAG GGC TTT CAT GTC CTG G |
| *Nanog* | TCT TCC TGG TCC CCA CAG TTT | GCA AGA ATA GTT CTC GGG ATG AA |
| *Jmjd1a* | CAG AGC AAA ATG GGG CAT AAA GG | GAC AAG GAT GTC TGT TTC AGG TC |
| *HP1* | CCA AGG AAG CCA ATG TCA AGT | GGA ATG CCA CGT TAG CCT TTC |
| *Dmnt1* | ATC CTG TGA AAG AGA ACC CTG T | CCG ATG CGA TAG GGC TCT G |
| *GAPDH* | AGG TCG GTG TGA ACG GAT TTG | TGT AGA CCA TGT AGT TGA GGT CA |
