# Supplementary figures and images for: Manipulation of Signaling Thresholds in “Engineered Stem Cell Niches” Identifies Design Criteria for Pluripotent Stem Cell Screens
Source: PLoS One. 2009 Jul 30;4(7):e6438. doi: 10.1371/journal.pone.0006438 (PMC2713412; doi:10.1371/journal.pone.0006438)

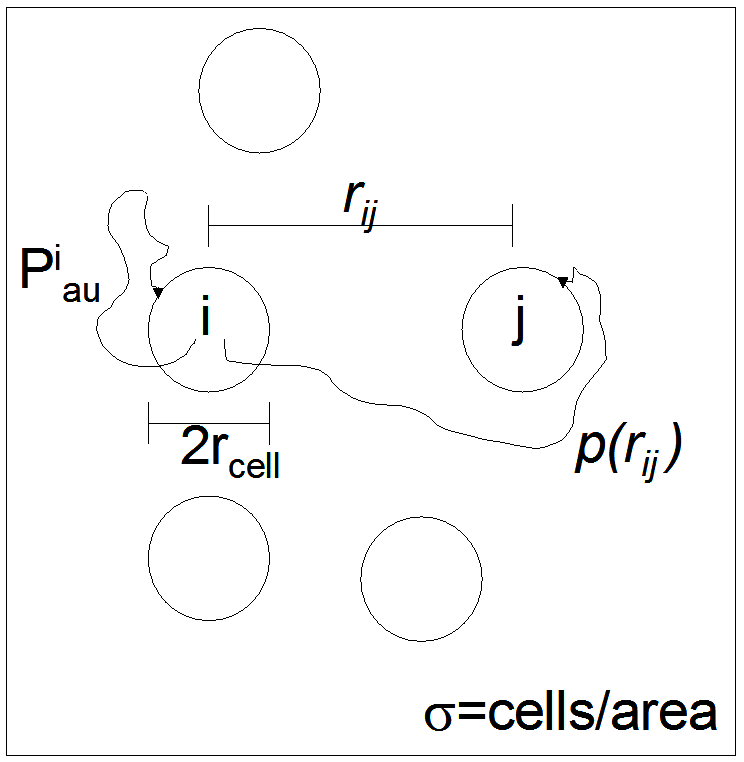

Supplement: Figure S1 — Schematic of the spatial parameters of the model. Complex number for cell i (Cin) is proportional to the sum of the probabilities of ligand capture by autocrine trajectories (Piau)and paracrine trajectories (Pipara) that are dependent of the radius of the cell (rcell). Pipara is determined by summing the paracrine contributions of each cell pair in the well p(rij)which has radial (rij) and cell density (σ) dependencies. (0.04 MB TIF) [file pone.0006438.s002.tif]

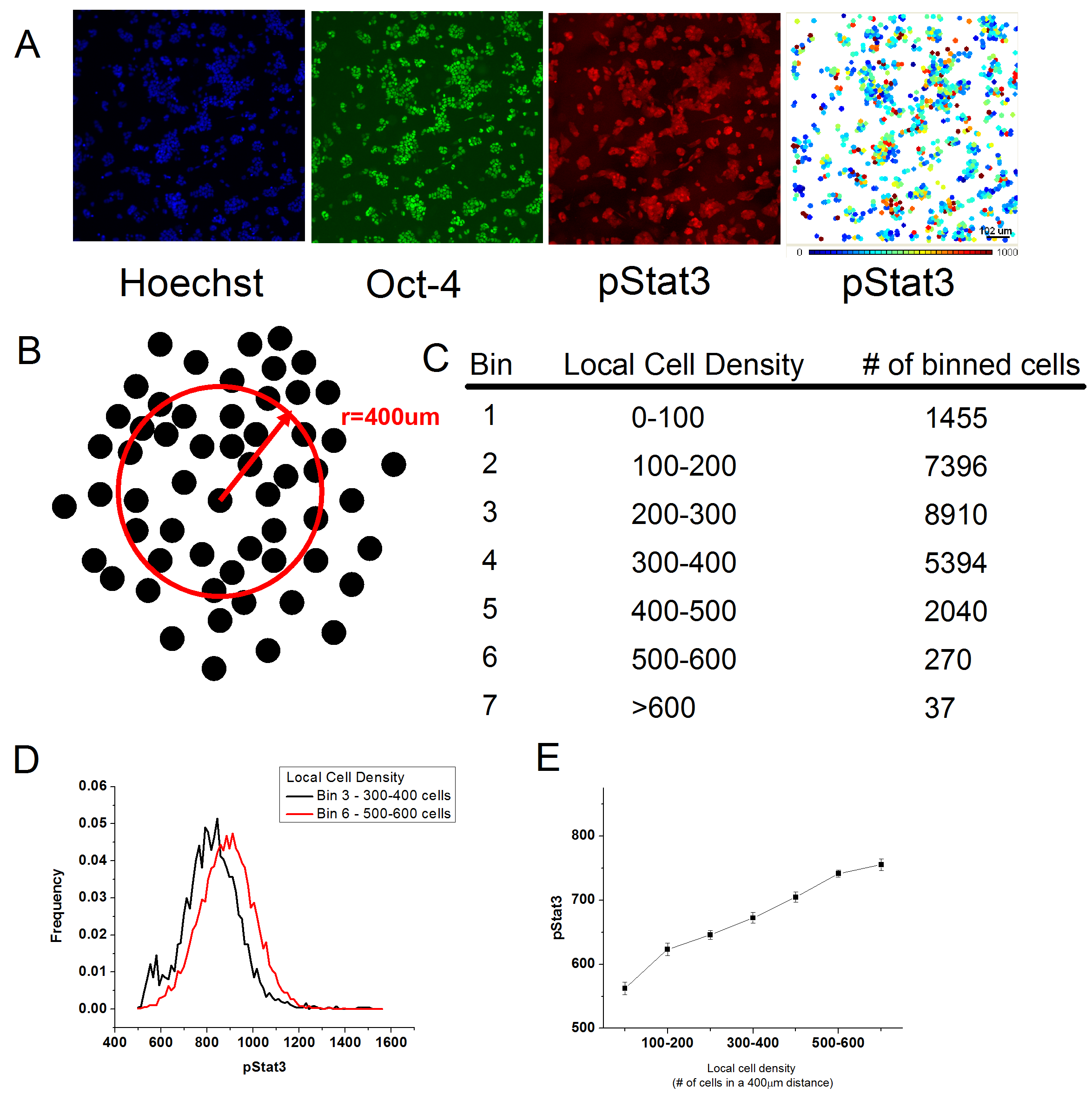

Supplement: Figure S2 — Development of the Neighbours Analysis algorithm. A) Photo-micrographs at 10X of non-patterned mESCs immunostained with Hoechst 33342, Oct-4, and pStat3 and the resulting heat-map constructed using the Python Imaging Library (PIL). Masks around individual nuclei were drawn using the Target Activation algorithm. B) Schematic of the Neighbours Analysis algorithm which counts the number of cells within a 400 µm radius which is dubbed local cell density. C) Example of binning for cells seeded at a density of 40,000 cells per well. D) Representative pStat3 histograms for cells after binning. E) Summary plot for data taken from the histograms to illustrate co-relations between pStat3 and local cell density. (1.54 MB TIF) [file pone.0006438.s003.tif]

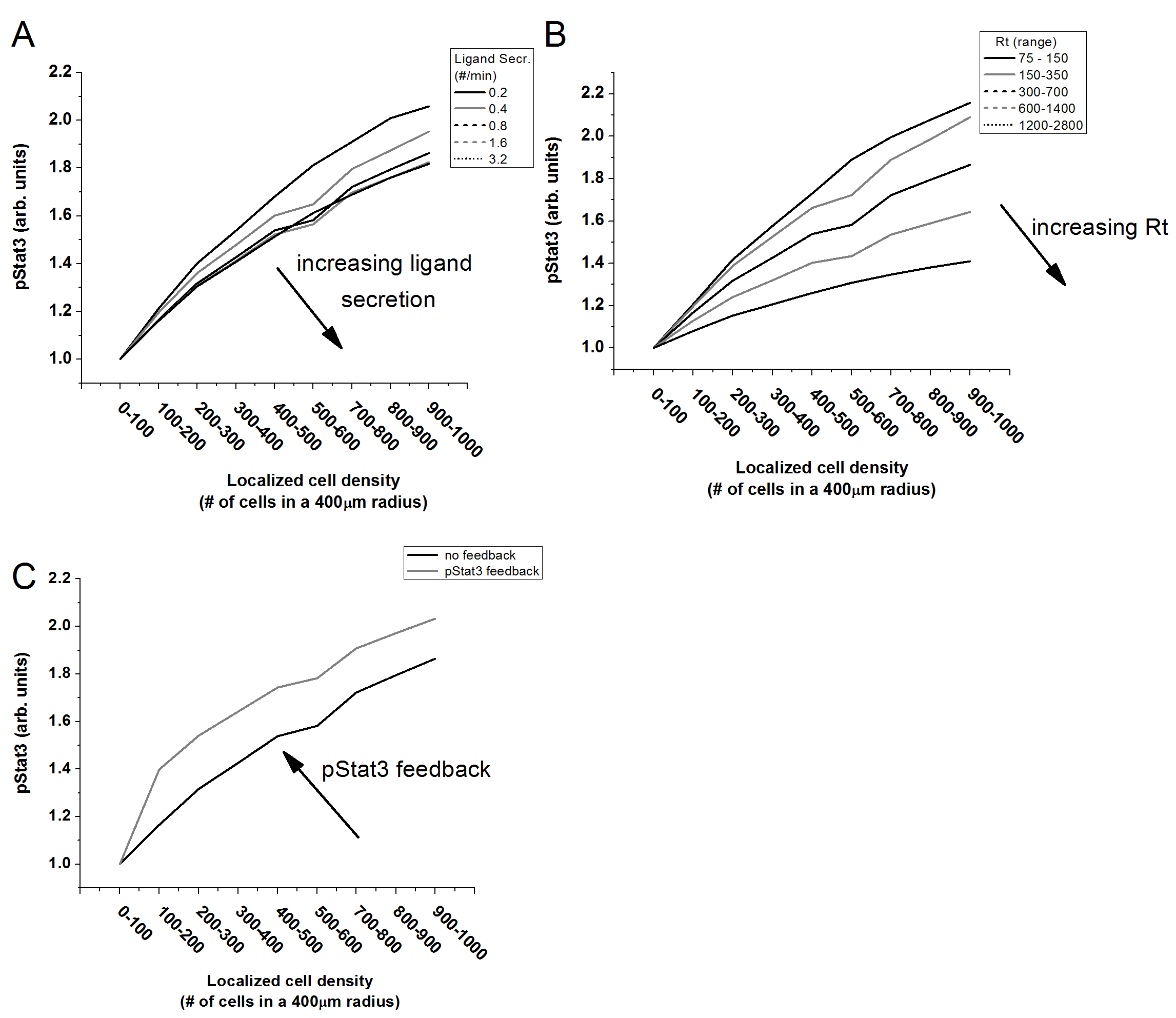

Supplement: Figure S3 — Theoretical predictions of how Jak-Stat pathway signaling components affect local signaling gradients. A) In these in silico experiments, the spatial arrangement of mESCs cultured in a single 96-well (approximately 35,000 cells) were inputted to the model. The predicted increase in pStat3 with increased localized cell density was computed while varying three parameters: endogenous ligand secretion, receptor number (Rt), and the presence of a positive feedback loop that increases total Stat3 (C). A) Predicted gradients in Stat3 as a function of increasing ligand secretion. According to model data, the relative changes in Stat3 that co-relate with localized cell density decrease moderately with increased ligand secretion. B) Predicted gradients in Stat3 as a function of increased receptor number (Rt). Increasing receptor number decreases the relative change in Stat3 with localized cell density. C) Predicted gradients in Stat3 in the presence of an auto-regulatory positive feedback loop that increases Stat3 in mESCs. This feedback loop has been previously described and modelled[18], [29]. The effect of this loop is to accentuate the effect of localized cell density on Stat3 gradients present in the culture. (0.40 MB TIF) [file pone.0006438.s004.tif]

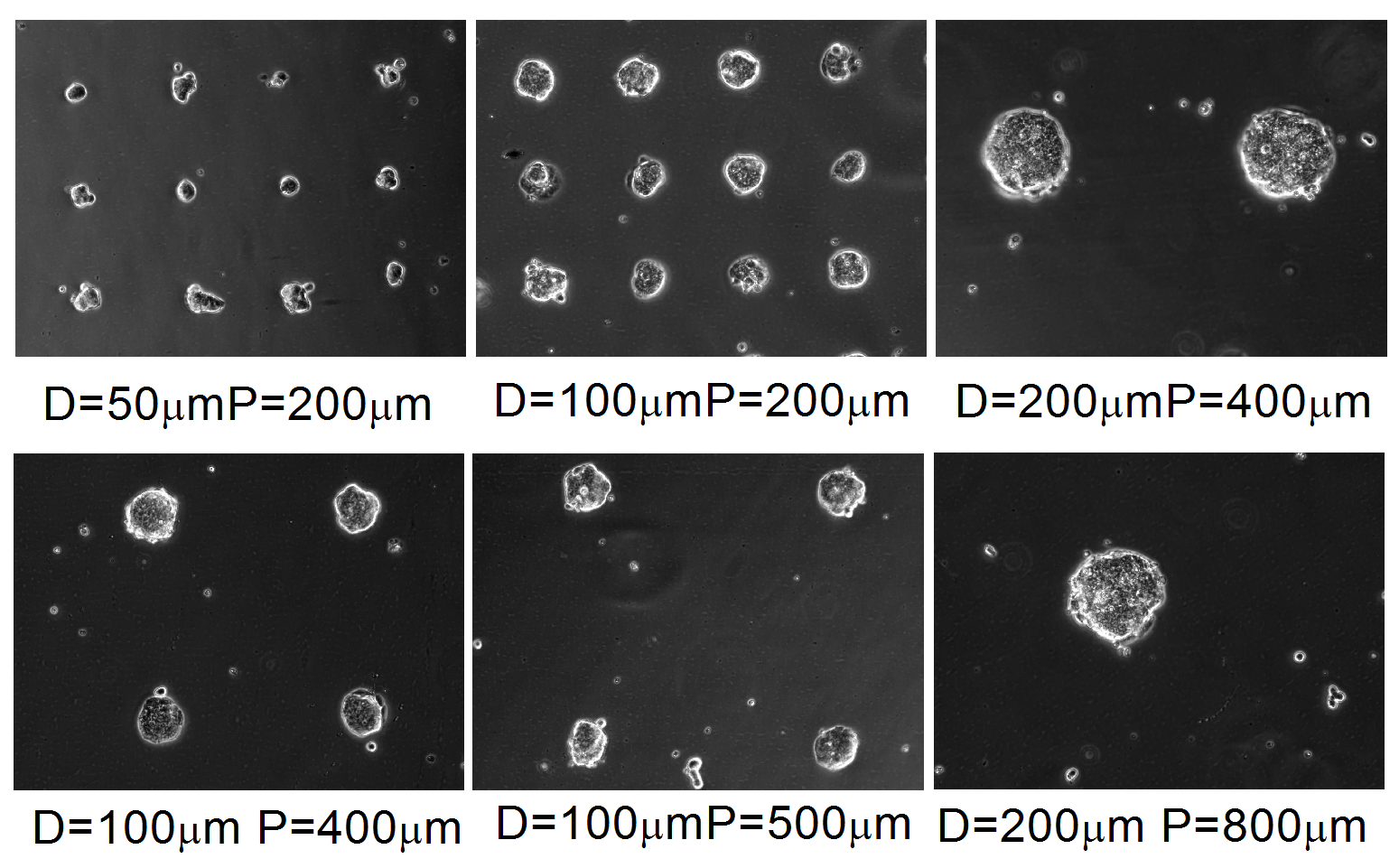

Supplement: Figure S4 — Bright field images of micro-patterned colonies. Bright field images of mESCs seeded in six different pattern types after 24 hours of culture in serum-free media without LIF. Scale-bar is 200 µm. (1.15 MB TIF) [file pone.0006438.s005.tif]

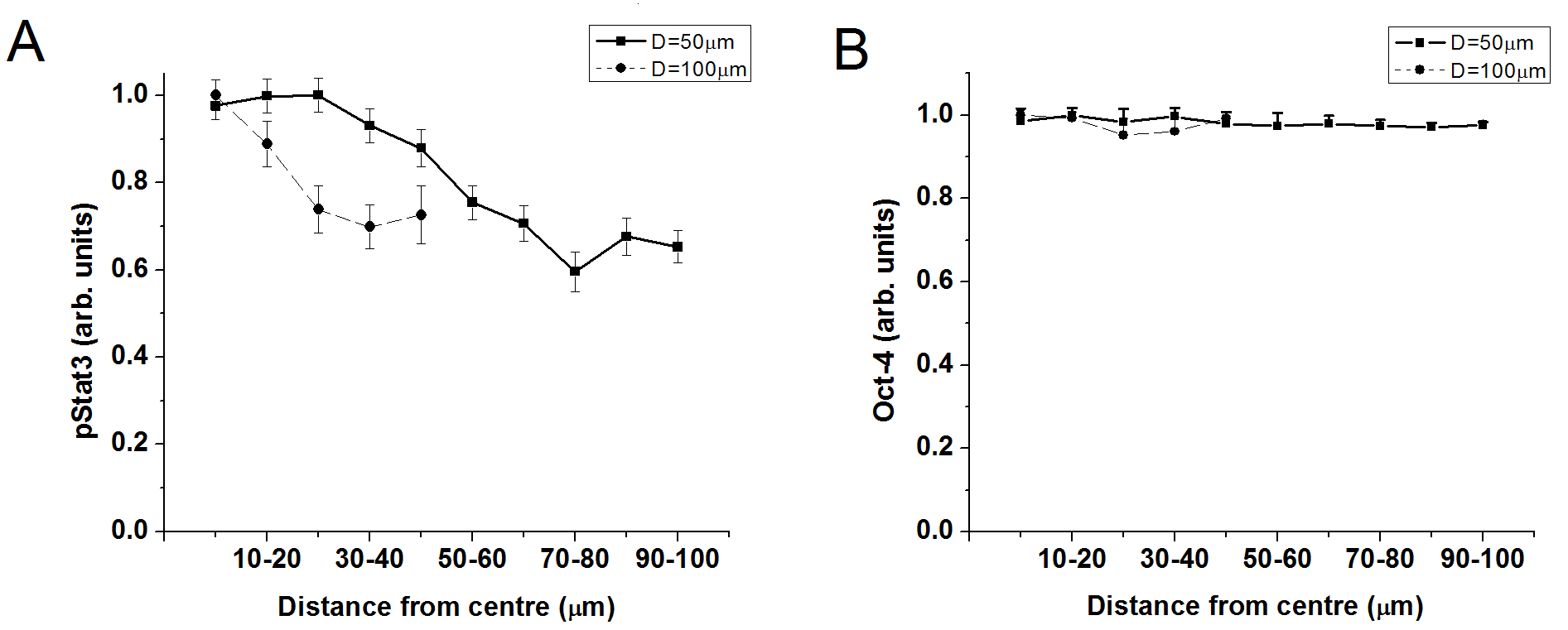

Supplement: Figure S5 — Radial organization of pStat3 within colonies. To reveal intra-colony variation in protein expression or signal activation, single-cell pStat3 and Oct-4 expression was plotted as a function of distance from the centre of the colony. mESCs were patterned in D = 50 µm, P = 200 µm and D = 100 µm, P = 200 µm arrangements. A) A radial dependence in pStat3 was observed in in both D = 50 µm and D = 100 µm colonies. B) No radial depense in Oct-4 was observed suggesting that the timeframe of the experiment (16 hours) was too short to reveal significant changes in Oct-4 expression. (0.10 MB TIF) [file pone.0006438.s006.tif]
